# TP53-Activated lncRNA GHRLOS Regulates Cell Proliferation, Invasion, and Apoptosis of Non-Small Cell Lung Cancer by Modulating the miR-346/APC Axis

**DOI:** 10.3389/fonc.2021.676202

**Published:** 2021-04-21

**Authors:** Ke Ren, Jinghui Sun, Lingling Liu, Yuping Yang, Honghui Li, Zhichao Wang, Jingzhu Deng, Min Hou, Jia Qiu, Wei Zhao

**Affiliations:** ^1^ School of Laboratory Medicine/Sichuan Provincial Engineering Laboratory for Prevention and Control Technology of Veterinary Drug Residue in Animal-origin Food, Chengdu Medical College, Chengdu, China; ^2^ Development and Regeneration Key Laboratory of Sichuan Province, Chengdu Medical College, Chengdu, China; ^3^ Department of Pulmonary and Critical Care Medicine, The First Affiliated Hospital of Chengdu Medical College, Chengdu, China; ^4^ Department of Refractive Surgery, Chengdu Aier Eye Hospital, Chengdu, China; ^5^ Department of Biomedical Sciences, City University of Hong Kong, Hong Kong, China

**Keywords:** NSCLC, TP53, lncRNA GHRLOS, miR-346, APC

## Abstract

Non-small cell lung cancer (NSCLC) is the main type of lung cancer with high mortality worldwide. To improve NSCLC therapy, the exploration of molecular mechanisms involved in NSCLC progression and identification of their potential therapy targeting is important. Long noncoding RNAs (lncRNAs) have shown important roles in regulating various tumors progression, including NSCLC. We found lncRNA GHRLOS was decreased in NSCLC cell lines and tissues which correlated with poor prognosis of NSCLC patients. However, the role and underlying mechanisms of lncRNA GHRLOS in NSCLC progression remains elusive. The expression of lncRNA GHRLOS was examined in NSCLC cell lines and biopsy specimens of patients with NSCLC by quantitative real time polymerase chain reaction (qRT-PCR). The effects of GHRLOS on proliferation, invasion and apoptosis of NSCLC cells were determined by both *in vitro* and *in vivo* experiments. The interaction between GHRLOS and TP53 was determined by dual-luciferase reporter assay and chromatin immunoprecipitation (ChIP) combined with qRT-PCR analysis. RNA immunoprecipitation (RIP) was conducted to validate the binding between GHRLOS and microRNA-346 (miR-346). Dual-luciferase reporter assays were also carried out to reveal the interaction between miR-346 and the 3’ untranslated region (3’UTR) of adenomatous polyposis coli (APC) mRNA.Our data demonstrated that overexpression of lncRNA GHRLOS suppressed cancer cell proliferation and invasion as well as promoted cell apoptosis by regulating the expression of CDK2, PCNA, E-cadherin, N-cadherin, Bax, and Bcl-2 in NSCLC cells. Moreover, lncRNA GHRLOS was upregulated by the binding of TP53 to the *GHRLOS* promoter. The binding target of lncRNA GHRLOS was identified to be miR-346. Impressively, overexpression of miR-346 promoted cell proliferation and invasion, as well as inhibited cell apoptosis, however, these effects can be blocked by overexpression of lncRNA GHRLOS both *in vitro* and *in vivo*. In summary, this study reveals lncRNA GHRLOS, upregulated by TP53, acts as a molecule sponge of miR-346 to cooperatively modulates expression of APC, a miR-346 target, and potentially inhibits NSCLC progression *via* TP53/lncRNA GHRLOS/miR-346/APC axis, which represents a novel pathway that could be useful in targeted therapy against NSCLC.

## Introduction

Lung cancer has been the leading cause of cancer-associated death globally (1–3). Non-small cell lung cancer (NSCLC) is the main sub-type of lung cancer (4–6), and its 5-year survival probability remains less than 15% as a result of recurrence and deficient treatment options (7, 8).To improve NSCLC therapy, it is critical to understand the molecular mechanisms involved in NSCLC progression. Therefore, a better interpretation of the potential molecular events in regulating NSCLC would offer novel prognostic and therapeutic biomarkers.

Long noncoding RNAs (lncRNAs) were once considered as “noise” in genomes, however, their functions in a variety of cancers, including NSCLC, are recently appreciated (9–14). It was reported that actin filament-associated protein 1 antisense RNA 1 (AFAP1-AS1) aggravates tumorigenesis and drug resistance in NSCLC (15–17). Overexpression of long non-coding RNA maternally expressed gene 3 (MEG3) resulted in increased apoptosis in NSCLC by regulating microRNA-7-5p (18). Small nucleolar RNA host gene 7 (SNHG7) is upregulated in NSCLC and indicates a poor prognosis. SNHG7 serves as a competitive endogenous RNA (ceRNA) by sponging miR-193b to increase expression of Fas apoptotic inhibitory molecule 2 (FAIM2) (19). Therefore, investigation of the role of lncRNAs in NSCLC could contribute to better treatment for NSCLC patients.

Ghrelin opposite strand/antisense RNA (GHRLOS) is the antisense strand of the ghrelin (*GHRL*) gene, which encodes active peptides such as ghrelin, a multifunctional peptide hormone involved in cancer development, insulin release, glucose metabolism, and gut motility (20–22). The level of lncRNA GHRLOS is reduced in human colorectal cancer tissues, and its high expression suggests a better prognosis (23). A recent study suggests that lncRNA GHRLOS is an inhibitor of cancer progression and may serve as a candidate biomarker of tumor metastasis and a prognostic indicator in colorectal cancer (24).

In cancer cells, lncRNAs were found to regulate gene expression at epigenetic, transcriptional and post-transcriptional levels by gene imprinting, histone modification, chromatin remodeling, transcriptional activation, transcriptional interference, nuclear transport and others (25). Emerging data suggests that lncRNAs work as sponges by binding competitively to microRNAs (miRNAs) and consequently repressing their function in lung cancer cells (26). Various miRNAs have been reported to play multiple roles in regulating tumor progression of NSCLC through the interaction with lncRNAs in many researches (27–29). However, the biofunction of miRNA-346, which dysregulated expression in NSCLC cells and tissues fund in this study, was still not clear. Accumulating evidence shows that lncRNA GHRLOS and miRNA-346 play important roles in cancers. However, the functions of lncRNA GHRLOS and miRNA-346 in NSCLC remains largely unknown.

As one of the well-known tumor suppressor, tumor protein p53 (TP53) has been reported to play important role in association with cancer progressions such as proliferation, invasion and metastasis, as well as resistance to chemo- or radio-therapy (30). However, TP53 also has mutant alleles in about 50% of all cancers (31), which seriously affects its function in tumor suppression. Beyond the loss of gene copies, the mutation of TP53 often occurs in the DNA-binding domain (32). These TP53 mutations lead to decreased ability that transactivate downstream genes, and dysregulation of target genes accelerates NSCLC development (33, 34). Although a number of lncRNAs were reported to be regulated by TP53 (35), whether TP53 modulates lncRNA GHRLOS in NSCLC is unknown.

Here, we explored the lncRNA GHRLOS involved molecular signal in NSCLC progression, and suggested lncRNA GHRLOS as a promising therapeutic target for NSCLC.

## Materials and Methods

### Specimens Collection and Overall Survival Analysis

A total of 134 pairs of NSCLC tissues and adjacent non-cancerous tissues were collected from the Department of pulmonary and critical care medicine in the First Affiliated Hospital of Chengdu Medical College (Chengdu, China). All participants signed an informed consent form. No patients were given chemotherapy and radiotherapy before surgery. The clinical samples were stored in liquid nitrogen before used for analysis. This study was approved by the Ethics Committee of the First Affiliated Hospital of Chengdu Medical College Chengdu. The overall survival rate was evaluated by Kaplan–Meier analysis according to lncRNA GHRLOS expression using Kaplan-Meier Plotter (36).

### H&E Staining

Briefly, the clinical samples were fixed in 4% paraformaldehyde, embedded in paraffin and sectioned similar to our previous study (16). After dewaxing in dimethylbenzene, the slides were rehydrated in graded ethanol. Then, they were stained with hematoxylin for 15 min, and with eosin for 3 min. Finally, the stained sections were subjected to dehydration and mounting. They were observed using a Carl Zeiss microscope (Axio Observer A1, Jena, Germany).

### Cell Culture

The pulmonary epithelial cell line BEAS-2B cells and NSCLC cell lines including A549, PC-9, NCI-H460, and NCI-H1975 cells were bought from the national biomedical experimental cell resource bank (Beijing, China). The cells were maintained in RPMI 1640 basic medium (Gibco/Thermo Fisher Scientific, Shanghai, China) containing 100 U/ml penicillin, 100 mg/ml streptomycin (Biological Industries, Hertzliya Pituach, Israel), and supplemented with 10% fetal bovine serum (Gibco/Thermo Fisher Scientific). They were placed in a humidified incubator with 5% CO_2_ at 37°C.

### Plasmids Construction and Interfering RNA Fragment Preparation

The Lv-GHRLOS contained full length of lncRNA GHRLOS (UCSC ID: ENST00000439539.3) for overexpression of GHRLOS, and Lv-sh-GHRLOS expressed 5’-GCUCCAUAAUGAACAUUGUUU-3’ for knockdown of GHRLOS whereas the control vector expressed 5’-AGA UCG ACG UGG CGU AAU CCA‐3’ (named Lv-sh-NC-lnc). Meanwhile, Lv-TP53 contained full length of TP53 (NCBI Reference Sequence: NM_000546.5) for overexpression of TP53, and Lv-sh-TP53 expressed 5’-GCACAGAGGAAGAGAAUCUUU-3’, and Lv-vector was used as a negative control. Lv-sh-APC expressed the sequences: 5’-CCUGCAAAUAGCAGAAAUAUU‐3’, and Lv-sh-NC expressed: 3′-ACGGUCAAA UUCCUCGAUAUC‐3’. Then, the GHRLOS promoter (-1500bp, +50bp) and its corresponding mutants (as illustrated in **Figure 3H**) were constructed into pmirGLO vector for luciferase reporter assays, and they were named pmirGLO-PF, pmirGLO-ΔE1, and pmirGLO-ΔE2. The full length of GHRLOS and its mutants were inserted into pmirGLO empty vector for dual luciferase reporter assays, and they were termed pmirGLO-GHRLOS-WT and pmirGLO-GHRLOS-mut, respectively. Similarly, the APC 3′-UTR and its mutants were constructed into pmirGLO-vector for dual luciferase reporter assays, and they were named pmirGLO-APC-3′UTR-WT and pmirGLO-APC-3′UTR-mut, respectively. In this study, The Mock, mimic, and inhibitor for miR-346 were constructed into lentiviruses. The Lv-Mock expressed 5’-UCACAACCUCCUAGAAAGAGUAGA-3’, and Lv-miR-346 mimic expressed 5′-UGUCUGCCCGCAUGCCUGCCUCU-3′, and Lv-miR-346 inhibitor expressed 5′-AGAGGCAGCGCGGGGCAGACA-3′. In this study, the lentivirus vectors were from Addgene (Watertown, MA, USA), and pGLO vector, also named pmirGLO, was bought from Promega (Madison, USA). Here, lentivirus was added into cells medium as our previous report (16).

### Quantitative Real Time PCR (qRT-PCR) Analysis

TRIzol reagent (Thermo Fisher Scientific) was applied to extract the total RNAs from clinical tissues or lung cell lines. 0.5 μg total RNA was prepared for reverse transcription into complementary DNA (cDNA) using SMART MMLV Reverse Transcriptase (Takara Bio, Inc., Dalian, Liaoning). Quantitative real-time PCR was performed to determine the gene expression in tissues and cells using AceQ qPCR SYBR GreenMaster Mix (Vazyme, Nanjing, China) on a CFX96 Real-Time PCR Detection System (Bio-Rad Laboratories, Inc., Hercules, USA). The primers were shown in **Table S1** and **Table S2** in **Supplementary Materials**.

### Western Blot

The tissues and cells were lysed with RIPA buffer to prepare proteins (Thermo Fisher Scientific). The 30 μg protein supernatant was separated by sodium dodecyl sulfate-polyacrylamide gel electrophoresis (SDS-PAGE), and transferred onto a nitrocellulose membrane (Bio-Rad, Hercules, CA, USA).The membranes were blocked with 5% no-fat milk for 0.5 h, and incubated with primary antibodies at 4°C overnight, then followed by incubating with the secondary antibodies at room temperature for 1 h. Finally, the protein bands on membranes were captured using a luminescence instrument (Tanon, Shanghai, China), and the gray density of protein bands were determined by Image J software (Media Cybernetics, Bethesda, MD). The primary antibodies were listed below, anti-APC (Cat. no. ab40778, abcam), anti-β-catenin (Cat. no. #9562, Cell Signal Technology), anti-Phospho-β-catenin (Cat. no. #2009, Cell Signal Technology), anti-GAPDH (Cat. no. #MB9231, Bioworld Technology). The secondary antibodies were bought from Bioworld, horseradish peroxidase (HRP)‐labeled goat anti‐rabbit or -mouse secondary antibody (Cat. no. # BS13278 or #BS12478, Shanghai, China). All other agents were purchased from Sigma (St. Louis, MO, USA).

### CCK-8 Assays and Colony Formation Assays

After transfection for 48 h, A549 and NCI-H460 cells were planted in a 96-well plate. At the indicated time, 10 μl CCK-8 solution (Sigma) was added into each well. After 1.5 h incubation, the OD value at 450 nm was measured using a microplate reader (Thermo Fisher Scientific). The experiments were conducted in quadruplicate and repeated three times.

In the cancer cell colony formation assay, cancer cells were infected with the indicated lentivirus for 48 h. Then, 1 × 10^3^ infected cells were planted into 6-well plates incubating for 14 days. The colonies were stained with 0.3% crystal violet for imaging using a light microscope. The experiments were conducted in quadruplicate and repeated three times.

### Trans-Well Assays

The lentivirus infected cancer cells (2× 10^4)^ were planted in the top chamber coated with matrigel (Cat. no. # 354230, Corning, Shanghai, China), and incubated in serum-free RPMI 1640 basic medium. The lower chamber was placed with FBS-containing medium. After 24 h, the invading cells in lower chamber were fixed with 4% formaldehyde, and stained with 0.3% crystal violet. The invading cells were counted under a light microscope (Carl Zeiss, Axio Observer A1). The experiments were conducted in quadruplicate and repeated three times.

### Apoptosis Assays

After infection for 48h, A549 and NCI-H460 cells were collected using trypsin. The cells were incubated with annexin V and propidium iodide (PI) (Cat. no. #C1062L, Beyotime) for 15 min. The apoptotic cells were measured on the Becton Dickinson flow cytometer (Becton Dickinson, USA). The cells both annexin V and PI positive staining indicated cells in necrosis (post-apoptotic necrosis or late apoptosis), the cells Annexin V positive but PI negative staining indicated cells in early apoptosis, as well as the cells both annexin V and PI negative staining indicated cells were healthy (37). The experiments were conducted in quadruplicate and repeated three times.

### Dual-Luciferase Reporter Assays

The cancer cells were seeded in 24-well plate at the density of 5× 10^4^ cells/well. The cells were infected according to experimental design in this study. Briefly, to determine the effect of TP53 on transcriptional activity of GHRLOS promoter, the cells were transfected with the lentivirus containing either the wild-type promoter (WT, also named pmirGLO-PF) or mutated GHRLOS promoters (pmirGLO-ΔE1 or pmirGLO-ΔE2). To investigate the reciprocal regulation between GHRLOS and miR-346, the cells were infected with lentivirus containing miR-346 mimic, inhibitor or Mock for 48 h, and transfected with wild type GHRLOS in pmirGLO vector (pmirGLO-GHRLOS-WT), mutated GHRLOS (pmirGLO-GHRLOS-mut), wild type of APC 3’UTR (APC 3’UTR-WT) or mutated APC 3’UTR (pmirGLO-APC-3’UTR-mut). After transfection for 48 h, luciferase activity was analyzed using the Dual-Luciferase Assay Kit on a GloMax 20/20 Luminometer (Promega). The experiments were conducted in quadruplicate and repeated three times.

### Subcellular Fractionation Assays

The cytoplasm and nucleus were separated using the PARIS Kit (Cat. no. # AM1921, Life Technologies, MA, USA). qRT-PCR analysis was conducted on the content of miR-346 and GHRLOS in nucleus or cytoplasm. GAPDH was used as a cytoplasmic control for GHRLOS whereas U6 was taken as a nuclear control. The experiments were repeated three times.

### Chromatin Immunoprecipitation Assays (ChIP) and RNA Immunoprecipitation Assay (RIP)

To determine the binding between TP53 and GHRLOS, ChIP assay was performed using the SimpleChIP^®^ Enzymatic Chromatin IP Kit (Cell Signaling Technology, #9002) according to manufacturer’s instructions. Briefly, cancer cells were treated with 4% formaldehyde and sequentially incubated with glycine to quench the reaction of formaldehyde fixation, after rinse with ice-cold PBS, the cells were collected using cell lysis buffer. The chromatin in the mixture is fragmented by partial digestion with Micrococcal Nuclease to obtain chromatin fragments of 1 to 5 nucleosome. The enzymatic fragmentation of chromatin was used immediately for immunoprecipitation. Anti-TP53 (Cat. no. ab1101, abcam) and anti-IgG (Cat. no. I4131, Sigma) were incubated with Dyna beads Protein G (Life Technologies). Then, the protein-DNA complexes were precipitated with an indicated antibody and protein G beads at 4°C for overnight. After reversal of protein-DNA cross-links, the DNA is purified using DNA purification spin columns. In addition, the possible contaminating RNAs and proteins in obtained DNA were removed by using RNase and proteinase K, respectively. Finally, the immunoprecipitated and purified DNA was detected using qRT-PCR analysis. The used primers for ChIP were listed as below:

GHRLOS, 5’-TTGGAGGCCCCTCCCACGAACCTTCTCCCAT-3’ (Forward), 5′-GAGGGGCTCAGCTCCCTGTGCATTC-3′ (Reverse); and 5’-GGCGCAGGGGCT GCCCCTCACCCAG-3’ (Forward), 5’-GCTCTGCTGGTCTCGCACGACTTCTG-3’ (Reverse). The experiments were repeated three times.

To investigate the interaction between GHRLOS and miR-346, RIP analysis was conducted using a Magna RIP™ RNA‐Binding Protein Immunoprecipation Kit (Millipore, Billerica, MA, USA). The cell lysates were incubated overnight at 4°C in RIP buffer containing magnetic beads conjugated anti-Ago- 2 (Cat. no. SAB4301150, Sigma) or anti-IgG as a control (Cat. no. I4131, Sigma). Before isolating the immunoprecipitated RNAs, the harvested magnetic beads, which enriched RNAs, were first treated with Proteinase K buffer at 4°C for 3 h. Finally, the content of GHRLOS and miR-346 in immunoprecipitated RNAs was examined by qRT-PCR (primers were listed in above section). The experiments were repeated three times.

### NSCLC Xenograft Model

To establish the NSCLC xenograft model, 5x106 lentivirus infected NCI-H460 cells were collected at 100 g × 5 min, and were inoculated into 6-week old BALB/c nude mice (Model Animal Research Center of Nanjing University, Nanjing, China). Then, the volume of tumor was calculated every 4 days for 28 days by using the formula: volume=length × width2/2. Finally, the mice were sacrificed on the 28th day according to the animal protocol approved by the Research Ethics Committee of Chengdu Medical College (ID: SYXK2020196).

### Statistical Analysis

The results were presented as mean ± standard deviation (S.D.). The data were analyzed using GraphPad Prism 6 (CA, USA) and SPSS software (version 19.0, SPSS Inc., NY, USA). Statistical significance was tested by Two-tailed Student’s *t*-test for two groups comparisons and one-way analysis of variance (ANOVA) test with post-hoc analysis contrasts for multi-groups comparisons. A *p* value <0.05 was considered significant.

## Results

### LncRNA GHRLOS Expression Was Decreased in NSCLC Cells and Tissues, and Correlated With Poor Prognosis of NSCLC Patients

**Figure 1:**
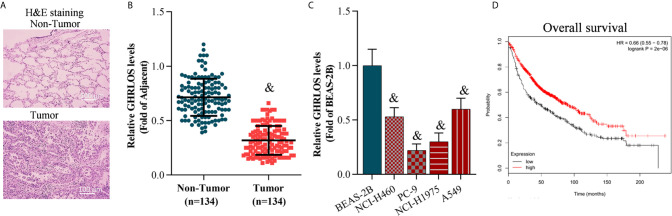
LncRNA GHRLOS expression decreased in lung tumor tissues and is clinically significant. **(A)** H&E staining of clinical lung tissue samples, including tumor tissues and non-tumor tissues (adjacent tissues). **(B)** qRT-PCR analysis of lncRNA GHRLOS expression in lung tissues, &*p*< 0.01 *vs.* non-tumor. **(C)** qRT-PCR analysis of lncRNA GHRLOS expression in human normal pulmonary epithelial cell line (BEAS-2B) and NSCLC cell lines, &*p*< 0.01 *vs.* BEAS-2B. **(D)** Kaplan–Meier analysis of overall survival in NSCLC patients based on lncRNA GHRLOS expression.

The NSCLC tissues and adjacent non-tumor tissues were collected and confirmed by H&E staining (**Figure 1A**). Quantitative RT-PCR was performed to evaluate the level of GHRLOS in these tissues. It was found that lncRNA GHRLOS was significantly downregulated in NSCLC tumors tissues and cancer cells compared with adjacent non-tumor tissues and the normal lung BEAS-2B cells (**Figures 1B, C**). The patients were separated into high- and low- lncRNA GHRLOS expression groups using median value as a cut point. Kaplan–Meier analysis was used to evaluate the overall survival in these patients following lncRNA GHRLOS expression. The results showed that high lncRNA GHRLOS expression was associated with better NSCLC prognosis (**Figure 1D**). These data indicated that lncRNA GHRLOS might function as a tumor suppressor in the progression of NSCLC.

### Overexpression of lncRNA GHRLOS Inhibited Cell Proliferation and Invasion and Increased Cell Apoptosis

**Figure 2:**
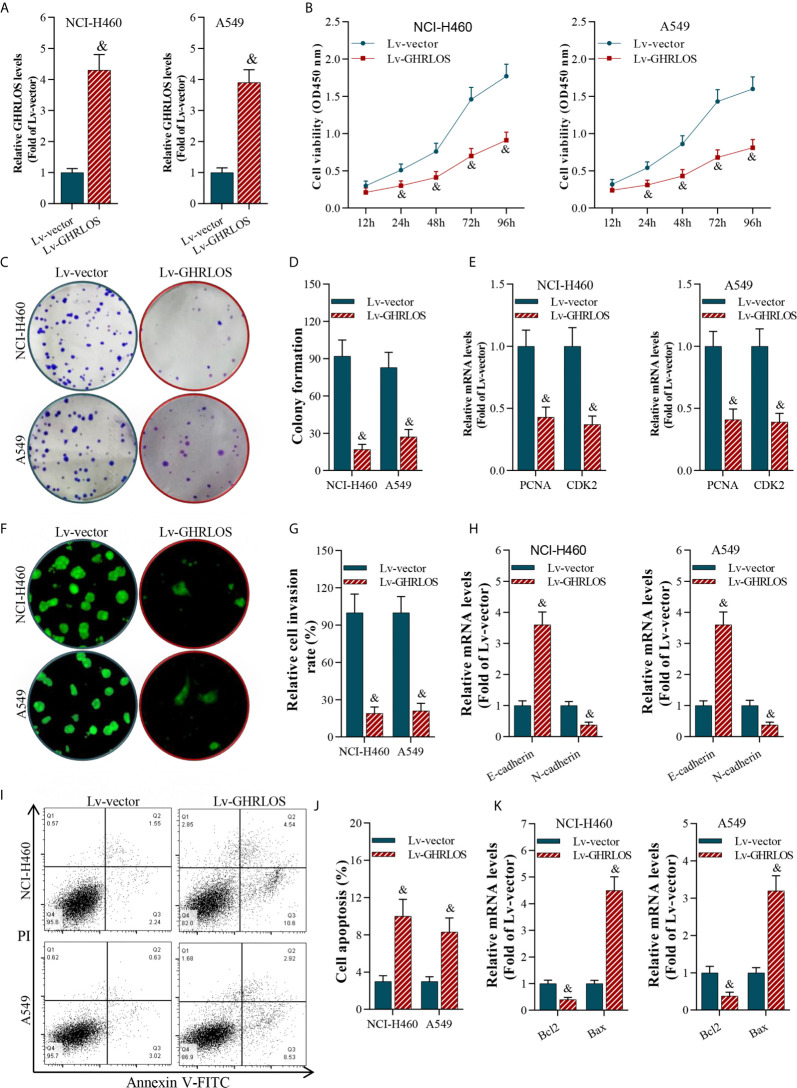
LncRNA GHRLOS inhibited NSCLC cell growth and invasion, and increased apoptosis. **(A)** qRT-PCR determination on lentivirus-induced overexpression of lncRNA GHRLOS at 48 h in A549 and NCI-H460 cells, &p< 0.01 *vs.* Lv-vector. **(B)** CCK-8 assay on the effect of lncRNA GHRLOS on cancer cell proliferation after lentivirus infection at the indicated time points, &p< 0.01 *vs.* Lv-vector. **(C, D)** Colony formation assay on the effect of lncRNA GHRLOS on growth of cancer cells after 14 days of culture, &p< 0.01 *vs.* Lv-vector. **(E)** qRT-PCR analysis on the expression of cell growth-associated genes, PCNA and CDK2, in A549 and NCI-H460 cells after overexpressed lncRNA GHRLOS for 48 h, &p< 0.01 *vs.* Lv-vector. **(F, G)** Trans-well assay analysis on the effect of lncRNA GHRLOS on the invasion of cancer cells, &p< 0.01 *vs.* Lv-vector. **(H)** qRT-PCR analysis on cell adhesion-related genes, including E-cadherin and N-cadherin, in A549 and NCI-H460 cells after infection for 48 h, &p< 0.01 *vs.* Lv-vector. **(I, J)** Annexin V-FITC/PI analysis on the effect of lncRNA GHRLOS on cancer cell apoptosis, &p< 0.01 *vs.* Lv-vector. **(K)** qRT-PCR analysis on the expression of cell apoptosis related genes, Bax and Bcl-2, in NSCLC cells infected by lentivirus for 48 h, &p< 0.01 *vs.* Lv-vector.

To investigate the role of lncRNA GHRLOS in cancer cell proliferation, invasion, and apoptosis, we overexpressed lncRNA GHRLOS in A549 and NCI-H460 cells by lentivirus infection (**Figure 2A**). Overexpression of lncRNA GHRLOS obviously inhibited A549 and NCI-H460 cell proliferation, as evidenced by CCK-8 and colony formation assays (**Figures 2B–D**). Overexpression of lncRNA GHRLOS significantly downregulated the expression of cell growth biomarkers, including PCNA and CDK2, in NSCLC cells (**Figure 2E**). lncRNA GHRLOS overexpression suppressed cancer cell invasion (**Figures 2F, G**) with downregulation of N-cadherin and upregulation of E-cadherin, that are epithelial-to-mesenchymal transition (EMT) associated biomarkers (**Figure 2H**). Finally, lncRNA GHRLOS overexpression markedly promoted A549 and NCI-H460 cell apoptosis (**Figures 2I, J**). Furthermore, lncRNA GHRLOS repressed Bcl-2 and upregulated Bax expression in cancer cell lines (**Figure 2K**). These results indicated that lncRNA GHRLOS functions as tumor suppressor by inhibiting proliferation and inducing apoptosis in NSCLC cells.

### TP53 Stimulated the Transcriptional Activation of lncRNAGHRLOS

**Figure 3:**
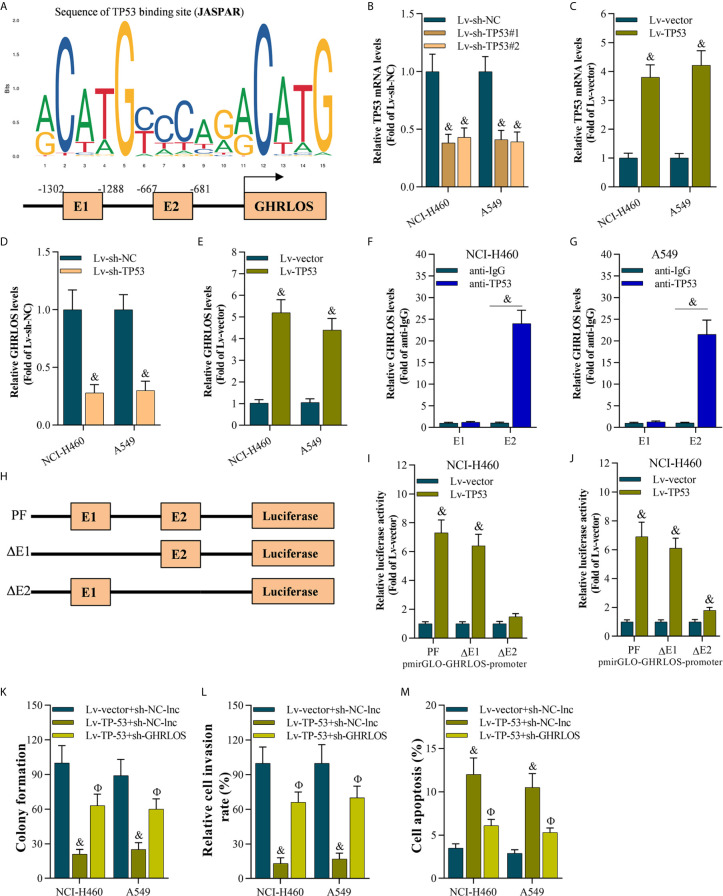
The TP53 activated lncRNA GHRLOS transcription in NSCLC cells. **(A)** The predicted binding motif of TP53 in the human lncRNA GHRLOS promoter based on analysis using JASPAR. **(B, C)** qRT-PCR analysis of TP53 expression after knockdown or overexpression of TP53 for 48 h in NSCLC cells, &p< 0.01 *vs.* Lv-sh-NC or Lv-vector. **(D, E)** qRT-PCR analysis on lncRNA GHRLOS expression after knockdown or overexpression of TP53 for 48 h in NSCLC cells, &p< 0.01 *vs.* Lv-sh-NC or Lv-vector. **(F, G)** ChIP assays evaluated TP53 binding sites, &p< 0.01 *vs.* anti-IgG. **(H)** Schematic diagram of plasmids used for luciferase reporter assays. **(I, J)** Dual luciferase reporter assays examined luciferase activity, &p< 0.01 *vs.* Lv-vector. **(K)** Colony formation assay on the growth of cancer cells infected with indicated lentivirus for 48 h followed by 14 days of culture, &p< 0.01 *vs.* Lv-vector, Фp< 0.01 *vs.* Lv-TP53. **(L)** Transwell assays on invasion of cancer cells 48 h after infection, &p< 0.01 *vs.* Lv-vector, Фp< 0.01 *vs.* Lv-TP53. **(M)** Annexin V-FITC/PI analysis on cancer cell apoptosis after infection for 48 h, &p< 0.01 *vs.* Lv-vector, Фp< 0.01 *vs.* Lv-TP53.

To explore the genes that regulated lncRNAGHRLOS in NSCLC cells, online prediction tools JASPAR (38) was used to predict the potential binding sites of the lncRNA GHRLOS promoter. TP53 was identified as the transcription factor with the highest binding potential based on the highest scores (**Figure 3A**) with several TP53 binding sites were predicted. In order to understand the relationship of TP53 and lncRNA GHRLOS, the expression of lncRNA GHRLOS was evaluated by qRT-PCR with inhibition of TP53. It was found that lncRNA GHRLOS expression was suppressed when TP53 was blocked, and upregulated when TP53 was overexpressed in NSCLC cells (**Figures 3B–E**). TP53 was found to bind to the GHRLOS promoter at “-667 to -681 (E2)” region, as evidenced by ChIP assays (**Figures 3F, G**). Dual luciferase reporter assays validated that the E2 site is the required binding region of TP53. As depicted in **Figure 3H**, the luciferase reporter plasmids, including PF (pGLO-PF), ΔE1, or ΔE2, were transfected into NSCLC cells. Luciferase assays revealed that TP53 failed to activate luciferase activity of the GHRLOS promoter without E2 region (**Figures 3H–J**). Collectively, TP53 is suggested to be a transcriptional factor in regulating lncRNA GHRLOS expression.

To evaluate the role of the TP53/lncRNA GHRLOS axis in cancer development, we overexpressed TP53 and silenced lncRNA GHRLOS expression in A549 and NCI-H460 cells. lncRNA GHRLOS knockdown significantly reversed TP53-mediated cell growth, invasion and apoptosis in NSCLC cells (**Figures 3K–M**).

### LncRNA GHRLOS Is a Molecular Sponge of miR-346 in NSCLC Cells

**Figure 4:**
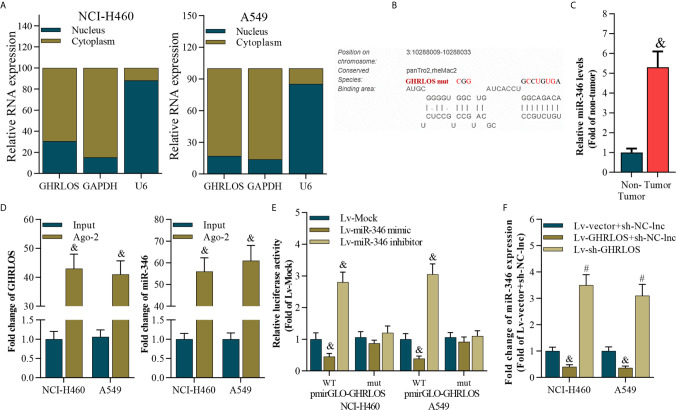
LncRNA GHRLOS is a molecular sponge of miR-346. **(A)** qRT-PCR analysis of subcellular localization of lncRNA GHRLOS. GAPDH was used as an internal cytoplasmic control, and U6 served as an internal nuclear control. **(B)** qRT-PCR analysis of miR-346 in clinical NSCLC tissues, &p< 0.01 *vs.* non-tumor. **(C)** The predicted binding site between lncRNA GHRLOS and miR-346 was obtained. **(D)** qRT-PCR analysis on the expression of lncRNA GHRLOS and miR-346 after anti-Ago2-mediated RIP assays in A549 and NCI-H460 cells, &p< 0.01 *vs.* input. **(E)** Dual luciferase reporter assay on the interaction between lncRNA GHRLOS and miR-346 after infection with Mock, miR-346 mimic, or miR-346 inhibitor for 48 h, &p< 0.01 *vs.* Lv-Mock. **(F)** qRT-PCR analysis of miR-346 after indicated infection for 48 h in A549 and NCI-H460 cells, &p< 0.01 *vs.* Lv-vector.

To analyze the subcellular location of lncRNA GHRLOS in NSCLC cells, the nucleus and cytoplasm of these cells were separated, and qRT-PCR were performed. Interestingly, lncRNA GHRLOS is primarily distributed in the cytoplasm of NSCLC cells (**Figure 4A**), suggesting that lncRNA GHRLOS could function as a ceRNA in NSCLC. The target miRNA of lncRNA GHRLOS was predicted using DIANA tools (39). It was found that miR-346 was a potential target of lncRNA GHRLOS and its binding sites were showed in **Figure 4B**. Furthermore, the expression of miR-346 was found to be elevated in NSCLC tissues (**Figure 4C**). Moreover, lncRNA GHRLOS interacted with miR-346, as evidenced by RIP assays (**Figure 4D**). Furthermore, dual luciferase reporter assays revealed that a miR-346 mimic efficiently repressed the luciferase activity of wild-type GHRLOS (pGLO-GHRLOS-WT), whereas a miR-346 mimic did not significantly affect the luciferase activity of mutant GHRLOS (pGLO-GHRLOS-mut) (**Figure 4E**). Finally, miR-346 expression was negatively regulated by lncRNA GHRLOS in NSCLC cells (**Figure 4F**). These results demonstrated that lncRNA GHRLOS is a molecular sponge of miR-346 in NSCLC cells.

### Overexpression of miR-346 Partly Reversed Overexpression of lncRNA GHRLOS-Mediated Cell Proliferation, Invasion, and Apoptosis Both *In Vitro* and *In Vivo*

**Figure 5:**
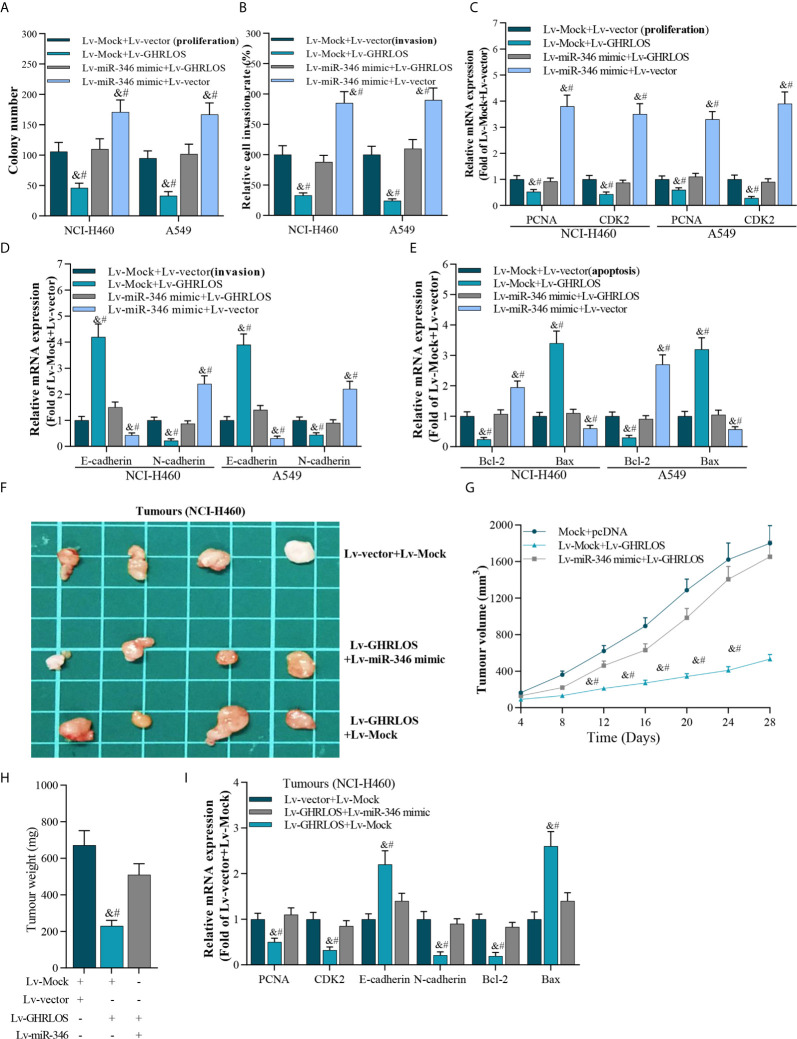
The interaction between lncRNA GHRLOS and miR-346 in cancer cell proliferation, invasion, and apoptosis. **(A)** Colony formation assay showed reciprocal suppression between lncRNA GHRLOS and miR-346 on the growth of transfected cancer cells after 14 days culture, &p< 0.01 *vs.* Lv-Mock+Lv-vector, #p< 0.01 *vs.* Lv-miR-346+Lv-GHRLOS. **(B)** Trans-well assays on invasion of lentivirus infected cancer cells, &p< 0.01 *vs.* Lv-Mock+Lv-vector, #p< 0.01 *vs.* Lv-miR-346+Lv-GHRLOS. **(C)** qRT-PCR on the expression of cell growth biomarkers, PCNA and CDK2, in A549 and NCI-H460 cells after infected with indicated lentivirus for 48 h, &p< 0.01 *vs.* Lv-Mock+Lv-vector, #p< 0.01 *vs.* Lv-miR-346+Lv-GHRLOS. **(D)** qRT-PCR on the expression of cell adhesion biomarkers, including E-cadherin and N-cadherin, in A549 and NCI-H460 cells, &p< 0.01 *vs.* Lv-Mock+Lv-vector, #p< 0.01 *vs.* Lv-miR-346+Lv-GHRLOS. **(E)** qRT-PCR analysis on the expression of cell apoptosis-associated genes, Bax and Bcl-2, in cancer cells after lentivirus infection for 48 h, &p< 0.01 *vs.* Lv-Mock+Lv-vector, #p< 0.01 *vs.* Lv-miR-346+Lv-GHRLOS. **(F)** Photo of tumors in nude mice after inoculation for 28 days. **(G)** The tumor volume of each group (n=6) measured at the indicated time points after inoculation of NCI-H460 cells. **(H)** The weight of tumors in nude mice after inoculation for 28 days, &p< 0.01 *vs.* Lv-Mock+Lv-vector, #p< 0.01 *vs.* Lv-miR-346+Lv-GHRLOS. **(I)** qRT-PCR analysis on the expression of PCNA, CDK2, E-cadherin, N-cadherin, Bcl-2, and Bax in xenografted tumors, &p< 0.01 *vs.* Lv-Mock+Lv-vector, #p< 0.01 *vs.* Lv-miR-346+Lv-GHRLOS.

The above data indicated that lncRNA GHRLOS could be a potential tumor suppressor and a molecular sponge of miR-346 in NSCLC cells. Colony formation and transwell assays demonstrated that miR-346 significantly increased cancer growth and cell invasion, but this effect was overturned by concomitant overexpression of lncRNA GHRLOS (**Figures 5A, B**). Similarly, lncRNA GHRLOS partly blocked miR-346 mimic-mediated regulation on the expression of PCNA, CDK2, E-cadherin, and N-cadherin in NSCLC cells (**Figures 5C, D**). In addition, lncRNA GHRLOS also partly abolished miR-346 mimic-mediated dysregulation on mRNA of Bcl-2 and Bax in A549 and NCI-H460 cells (**Figure 5E**).

The reciprocal inhibition between lncRNA GHRLOS and miR-346 was also investigated *in vivo*. LncRNA GHRLOS overexpression significantly inhibited tumor growth (**Figures 5F, G**) and decreased tumor weight in nude mice (**Figure 5H**). However, miR-346 overexpression markedly reversed lncRNA GHRLOS-induced suppression on tumor volume and weight (**Figures 5F–H**). Profoundly, miR-346 overexpression attenuated changes in gene expression induced by lncRNA GHRLOS, including PCNA, CDK2, E-cadherin, and N-cadherin, Bcl-2, and Bax (**Figure 5I**). These data showed that lncRNA GHRLOS and miR-346 play reciprocally modulated tumor suppressor and oncogenic roles, respectively, in NSCLC cells.

### APC Is a Direct Target of miR-346 and Is Regulated by Interaction Between lncRNA GHRLOS and miR-346

**Figure 6:**
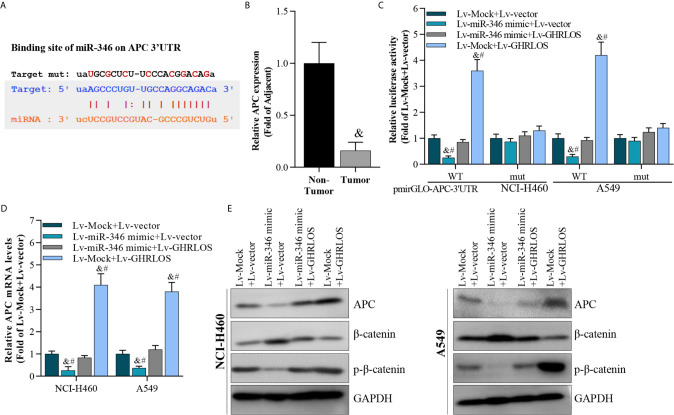
APC was a target of miR-346 and regulated by interaction of lncRNA GHRLOS and miR-346. **(A)** Predicted binding site of miR-346 in the APC 3’UTR (STARBASE database). **(B)** qRT-PCR analysis on APC expression in NSCLC tissues, &p< 0.01 *vs.* non-tumor. **(C)** Dual luciferase reporter assay on luciferase activity of APC 3’UTR, &p< 0.01 *vs.* Lv-Mock+Lv-vector, #p< 0.01 *vs.* Lv-miR-346+Lv-GHRLOS. **(D)** qRT-PCR analysis on APC expression in cancer cells infected with lentivirus for 48 h, &p< 0.01 *vs.* Lv-Mock+Lv-vector, #p< 0.01 *vs.* Lv-miR-346+Lv-GHRLOS. **(E)** Western blot analysis on APC expression in cancer cells after lentivirus infection for 48 h.

It was demonstrated that APC is a suppressor of the canonical Wnt/β-catenin pathway, which increases the transcriptional activation of oncogenes in NSCLC (40). Here, APC was identified to be a target of miR-346 by STARBASE online tools (41) (**Figure 6A**). We found that the expression of APC reduced in NSCLC tissues (**Figure 6B**). Dual luciferase reporter assays revealed that miR-346 inhibited the luciferase activity of APC 3’UTR in NSCLC cells, whereas miR-346-induced inhibition is significantly alleviated by lncRNA GHRLOS (**Figure 6C**). Moreover, lncRNA GHRLOS elevated the luciferase activity of APC 3’UTR in A549 and NCI-H460 cells, whereas the lncRNA GHRLOS-induced elevation was significantly reversed by miR-346 overexpression (**Figure 6C**). Similarly, lncRNA GHRLOS significantly reversed the effects of miR-346 on the expression of APC mRNA and protein, as demonstrated by qRT-PCR and Western blot (**Figures 6D, E**). These findings showed that APC is a direct target of miR-346, and its expression is modulated by the interaction between lncRNA GHRLOS and miR-346.

### Knockdown of APC Blocked miR-346 Inhibitor- or lncRNA GHRLOS Overexpression-Induced Expression of Genes Involved in the Growth, Invasion, and Apoptosis of NSCLC Cells

**Figure 7:**
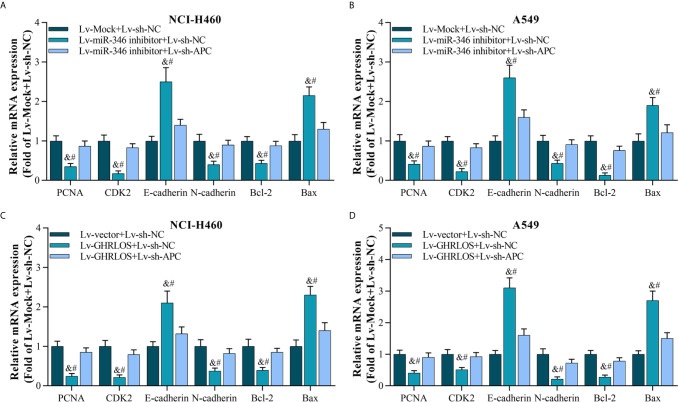
Silencing APC blocked miR-346 knockdown- or lncRNA GHRLOS overexpression-controlled gene expression on biomarkers of cell proliferation, invasion, and apoptosis. **(A, B)** qRT-PCR analysis on gene expression after lentivirus infection for 48 h in A549 and NCI-H460 cells, &*p*< 0.01 *vs.*Lv-Mock+Lv-sh-NC, #*p*< 0.01 *vs.*Lv-miR-346+Lv-sh-APC. **(C, D)** qRT-PCR analysis on gene expression in NSCLC cells with lentivirus infection for 48 h, &*p*< 0.01 *vs.*Lv-Mock+Lv-sh-NC, #*p*< 0.01 *vs.*Lv-miR-346+Lv-sh-APC.

As the downstream molecule of lncRNA GHRLOS/miR-346 axis, APC was interfered in NSCLC cells, and its effect was evaluated. The data showed that inhibition of APC expression blocks miR-346-induced genes dysregulation, including PCNA, CDK2, E-cadherin, N-cadherin, Bcl-2, and Bax (**Figures 7A, B**). Moreover, downregulation of APC also abolished the regulation of gene expression mediated by GHRLOS (**Figures 7C, D**). Therefore, the role of lncRNA GHRLOS and miR-346 in regulating cell proliferation, invasion, and apoptosis might require APC participation.

### APC Expression Is Regulated by TP53-Mediated lncRNA GHRLOS and miR-346

**Figure 8:**
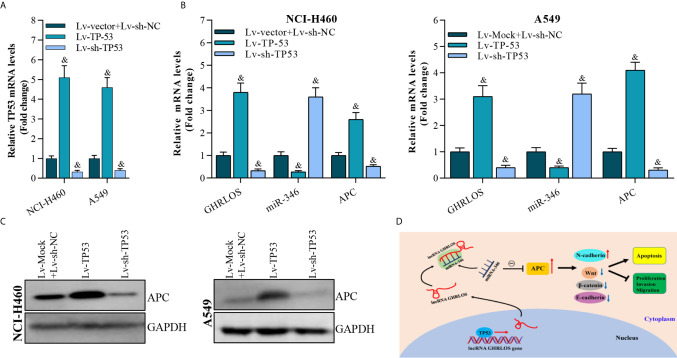
APC expression is modulated by overexpression or interference with TP53 expression. **(A)** qRT-PCR analysis on TP53 expression after overexpressed- or knockdown-TP53 for 48 h, &p< 0.01 *vs.* Lv-vector. **(B)** qRT-PCR analysis on APC, lncRNA GHRLOS, and miR-346 expression after indicated lentivirus infection for 48 hr in A549 and NCI-H460 cells, &p< 0.01 *vs.* Lv-vector. **(C)** Western blot analysis on APC expression in A549 and NCI-H460 cells with indicated infection. **(D)** Schematic presentation of molecular mechanism of lncRNA GHRLOS mediated cell proliferation, invasion and apoptosis in NSCLC.

To determine whether TP-53 was required for the regulation of APC expression in NSCLC cells, TP53 was overexpressed or knocked down by lentivirus infection (**Figure 8A**). qRT-PCR analysis demonstrated that TP53 overexpression upregulated the expression of both lncRNA GHRLOS and APC but repressed the expression of miR-346 (**Figure 8B**). Most impressively, APC expression was increased in response to TP53. In contrast, APC expression was decreased by knockdown of TP53 (**Figure 8C**). These results suggest that TP53 controls lncRNA GHRLOS- and miR-346-regulated APC expression.

Taken together, these results suggest that by competitively absorbing miR-346, lncRNA GHRLOS upregulates APC and further regulates cancer cell proliferation, invasion, and apoptosis in NSCLC (**Figure 8D**).

## Discussion

NSCLC is the most common type of lung cancer worldwide (1, 3–6). Increasing evidence has demonstrated that lncRNAs play essential roles in NSCLC tumorigenesis and progression. Our previous works showed that AFAP1-AS1 (16), DHRS4-AS1 (42) and KTN1-AS1 (43) participate in NSCLC progression *via* regulating diverse molecular mechanisms involved in cancer cell behaviors. Here, we reported a new NSCLC suppressor lncRNA, lncRNA GHRLOS. We found that lncRNA GHRLOS was downregulated in NSCLC tissues and cells, and its downregulation predicts a poor overall survival in patients. LncRNA GHRLOS significantly inhibited cell proliferation and invasion and increased apoptosis in NSCLC cells. These findings demonstrate that lncRNA GHRLOS exerts tumor inhibitory activity in the development of NSCLC, suggesting that overexpression of lncRNA GHRLOS could be used for treatment in patients with NSCLC.

A number of studies have revealed that lncRNA are regulated by TP53, a well-known transcription factor and tumor suppressor (35). We found that TP53 activates the transcription of lncRNA GHRLOS by binding to its promoter. In contrast, interruption of TP53 expression decreases lncRNA GHRLOS level in NSLC cells. Therefore, the downregulation of lncRNA GHRLOS is most likely caused by loss of DNA-binding domain of TP53 gene, which are common events in NSCLC. TP53 is encoded by the *TP53* gene, and it is the most frequently mutated gene in human cancers of many types mutations are common in NSCLC, and *TP53* mutations have been reported associate with poor prognosis of NSCLC patients (44, 45). Whether the occurrence of *TP53* gene mutation could affect the binding of TP53 with *GHRLOS* promoter, and hence inducing the loss of function of lncRNA GHRLOS in inhibiting the progression of NSCLC still required to verified. Therefore, make further efforts to estimate the influence of different *TP53* gene mutation on the binding with the promoter of gene encoding lncRNA, like GHROS, and explore the underlying regulatory mechanism in NSCLC progression are urgently needed.

In this study, it was found that lncRNA GHRLOS was mainly located in the cytoplasm of NSCLC cells, and lncRNA GHRLOS acts as a molecular sponge of miR-346. Previous studies have demonstrated that miR-346 may be regulated by lncRNA NBAT1 (46), DGCR5 (47), and circFBLIM1 (48) in cancers. Moreover, miR-346 facilitates NSCLC growth and metastasis and inhibits cell apoptosis *via* controlling XPC/ERK/Snail/E-cadherin signaling pathway (49). Consistent with these studies, our data demonstrated that miR-346 is overexpressed in NSCLC tissues, and miR-346 regulates the mRNA expression of PCNA, CDK2, E-cadherin, N-cadherin, Bcl-2, and Bax in NSCLC cells. These findings demonstrated that lncRNA GHRLOS and miR-346 reciprocally controlled the activities of tumor suppressor and oncogenes, respectively. However, whether other lncRNAs are involved in the regulation of miR-346 in NSCLC is not addressed in this study.

APC is a negative regulator of Wnt/β-catenin signaling, which promotes the transcriptional activation of oncogenes in cancers (40, 50–52). Here, our results showed that TP53 significantly increased APC expression by regulating lncRNA GHRLOS/miR-346 pathway in NSCLC cells. Jaiswal *et al.* reported that phosphorylated p53 can up-regulate the APC promoter activity with other transcriptional factors through direct binding (53). Therefore, TP53 might directly or indirectly regulate APC, and lncRNA GHRLOS/miR-346 axis might be an indirect pathway for TP53 to regulate APC.

In summary, we identified a novel transcription factor-mediated lncRNA-miRNA-mRNA axis in NSCLC cells in this study. The downregulation of lncRNA GHRLOS caused by TP53 mutation not only correlates with poor clinical outcome, but also promotes cancer progression of NSCLC. TP53 regulates APC expression through lncRNA GHRLOS/miR-346 axis. Thus, the components of the TP53/lncRNA GHRLOS/miR-346/APC signaling pathway could represent novel targets for NSCLC therapies.

## Data Availability Statement

The original contributions presented in the study are included in the article/**Supplementary Material**. Further inquiries can be directed to the corresponding author.

## Ethics Statement

The studies involving human participants were reviewed and approved by the ethics committee of first affiliated hospital of Chengdu Medical College. The patients/participants provided their written informed consent to participate in this study. The animal study was reviewed and approved by the Ethics Committee of Animal Experiments of Chengdu Medical College.

## Author Contributions

WZ designed this research. KR and JS performed experiments and drafted this manuscript. YY and LL participated in most of the experiments, collected tissues samples, and analyzed clinical data. HL, ZW, JD, MH, and JQ assisted with part of cell and animal experiments. WZ edited the manuscript. All authors contributed to the article and approved the submitted version.

## Funding

This work was supported by the National Natural Science Foundation of China (81602636 and 31800154), the Nanjing medical science and technology development project (ZKX15049), the Key project of science of Sichuan Education Department (18ZA0164), the Natural Science Foundation of Chengdu Medical College (CYZ18-01, CYZ18-04), the Technology Innovation R&D Project of Chengdu Science and Technology Bureau (2018-YFYF-00158-SN), the Open fund of Development and Regeneration Key Laboratory of Sichuan Province (SYS18-08, SYS20-07), the Natural Science Foundation of Laboratory Medicine School in Chengdu Medical College (JYZK201701) and the Miaozi project of science and Technology Department of Sichuan Province (2018RZ0095).

## Conflict of Interest

The authors declare that the research was conducted in the absence of any commercial or financial relationships that could be construed as a potential conflict of interest.

## Abbreviations

NSCLC, Non-small cell lung cancer; GHRLOS, Ghrelin opposite strand/antisense RNA; TP53, Tumor protein p53; APC, Adenomatous polyposis coli; lncRNAs, long non-coding RNAs; miRNA, microRNA; ChIP, chromatin immunoprecipitation; RIP, RNA Binding Protein Immunoprecipitation; H&E, Hematoxylin and eosin; CCK-8, Cell counting kit-8; CDK2, Cyclin-dependent kinase 2; PCNA, Proliferating cell nuclear antigen; qRT-PCR, Quantitative polymerase chain reaction; UTR, Untranslated region.
