# Supplementary material for: TP53-Activated lncRNA GHRLOS Regulates Cell Proliferation, Invasion, and Apoptosis of Non-Small Cell Lung Cancer by Modulating the miR-346/APC Axis
Source: Front Oncol. 2021 Apr 21;11:676202. doi: 10.3389/fonc.2021.676202 (PMC8097184; doi:10.3389/fonc.2021.676202)
Supplement: Supplementary file 1 [file DataSheet_1.pdf]

Fig. S1 A

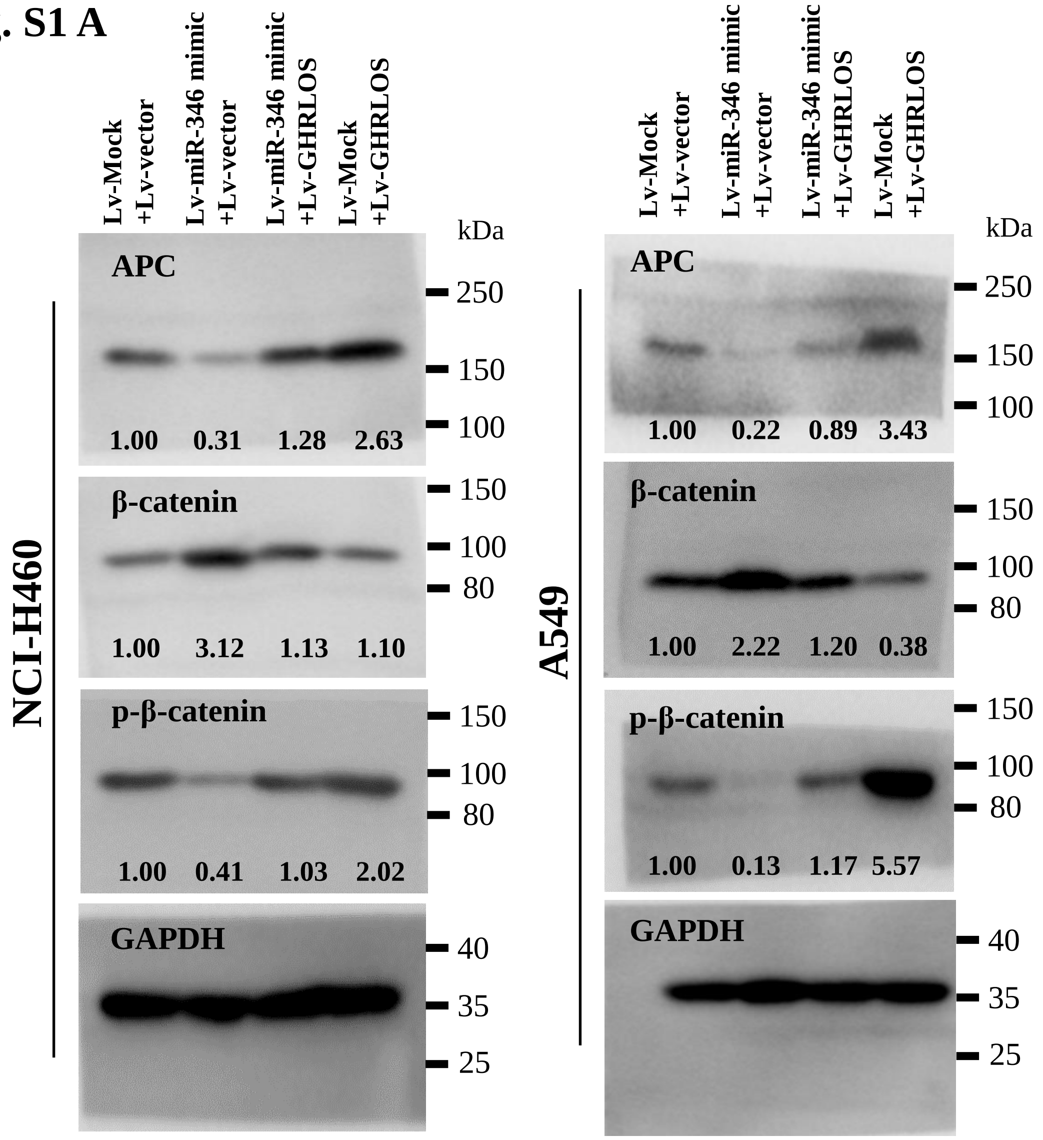

Fig. S1 A. The Original and crude Western blot images with molecular weight markers and relative integrated density. Fig. S1 A related to Fig. 6E.

Fig. S1 B

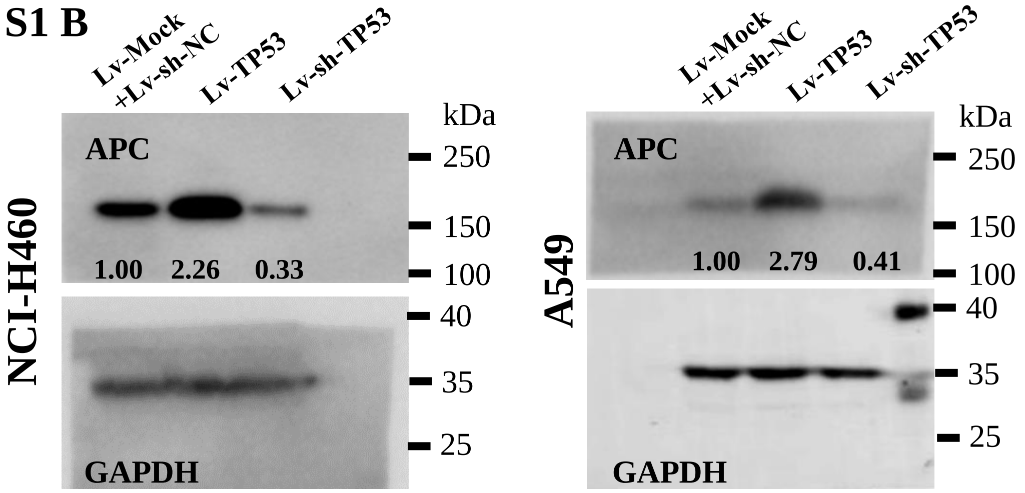

Fig. S1 B. The Original and crude Western blot images with molecular weight markers and relative integrated density. Fig. S1 B related to Fig. 8C.
