# Supplementary material for: TP53-Activated lncRNA GHRLOS Regulates Cell Proliferation, Invasion, and Apoptosis of Non-Small Cell Lung Cancer by Modulating the miR-346/APC Axis
Source: Front Oncol. 2021 Apr 21;11:676202. doi: 10.3389/fonc.2021.676202 (PMC8097184; doi:10.3389/fonc.2021.676202)
Supplement: Supplementary file 2 [file DataSheet_2.pdf]

Table S1. Primers used for Quantitative real time PCR

Gene	Forward （5’-3’）	Reverse （5’-3’）
GHRLOS	TGGAAACTCCCCTAGCCACA	GCATCTCTCCTCTGTTCCGT
PCNA	AACCGGTTACTGAGGGCGAG	AAAGTCTAGCTGGTTTCGGCT
CDK2	CTGCATCTTTGCTGAGATGGTGAC	GAAACTTGGCTTGTAATCAGGCA
E-cadherin	ATGCTGATGCCCCCAATACC	TGCCATCGTTGTTCACTGGA
N-cadherin	GAGGCTTCTGGTGAAATCGC	AATCTGCAGGCTCACTGCTC
Bcl-2	CCTGCTTCTTGAGACC	TGCTATTGTTGGGCAG
Bax	CAGAACCATCATGGGCTGGA	CAGGGACATCAGTCGCTTCA
TP53	AAGTCTAGAGCCACCGTCCA	CAGTCTGGCTGCCAATCCA
APC	ACAGAATGGAGGTGCTGCC	CTTCAGTGCCTCAACTTGCT
GAPDH	GAAGGCTGGGGCTCATTTG	AGGGGCCATCCACAGTCTTC
miR-346	TGTCTGCCCGCATGCCT	GTGCAGGGTCCGAGG
U6	AACGAGACGACGACAGAC	GCAAATTCGTGAAGCGTTCCA TA

Table S2. Primers used for Reverse Transcription PCR

Gene	Primer Sequence （5’-3’）
miR-346	GTCGTATCCAGTGCAGGGTCCGAGGTATTCGCACTGGATAGAGGC
U6	GTCGTATCCAGTGCAGGGTCCGAGGTATTCGCACTGGATACGACAAATATG
