# Supplementary figures and images for: TP53-Activated lncRNA GHRLOS Regulates Cell Proliferation, Invasion, and Apoptosis of Non-Small Cell Lung Cancer by Modulating the miR-346/APC Axis
Source: Front Oncol. 2021 Apr 21;11:676202. doi: 10.3389/fonc.2021.676202 (PMC8097184; doi:10.3389/fonc.2021.676202)

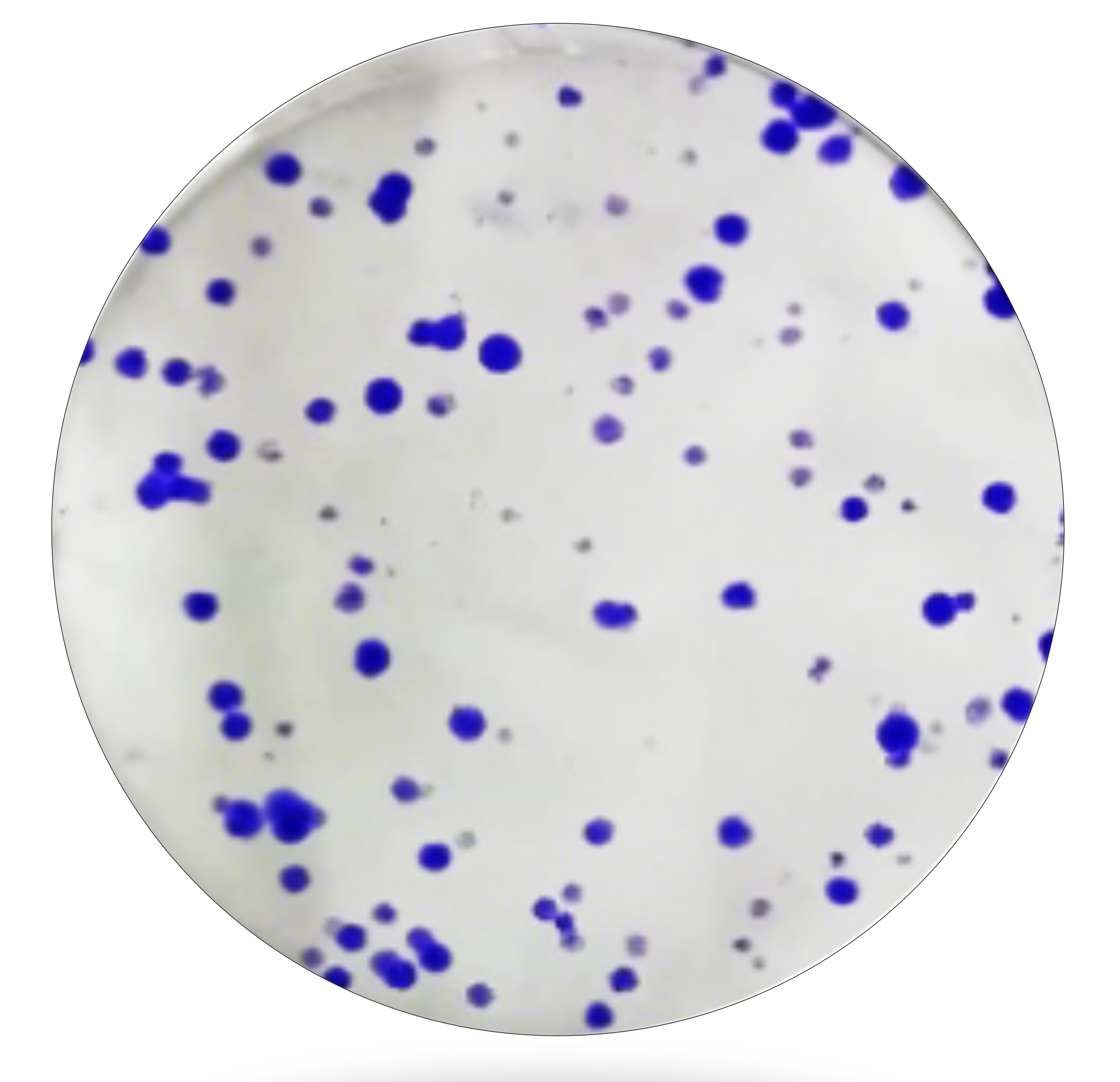

Supplement: Supplementary file 3 [file Image_1.tif]

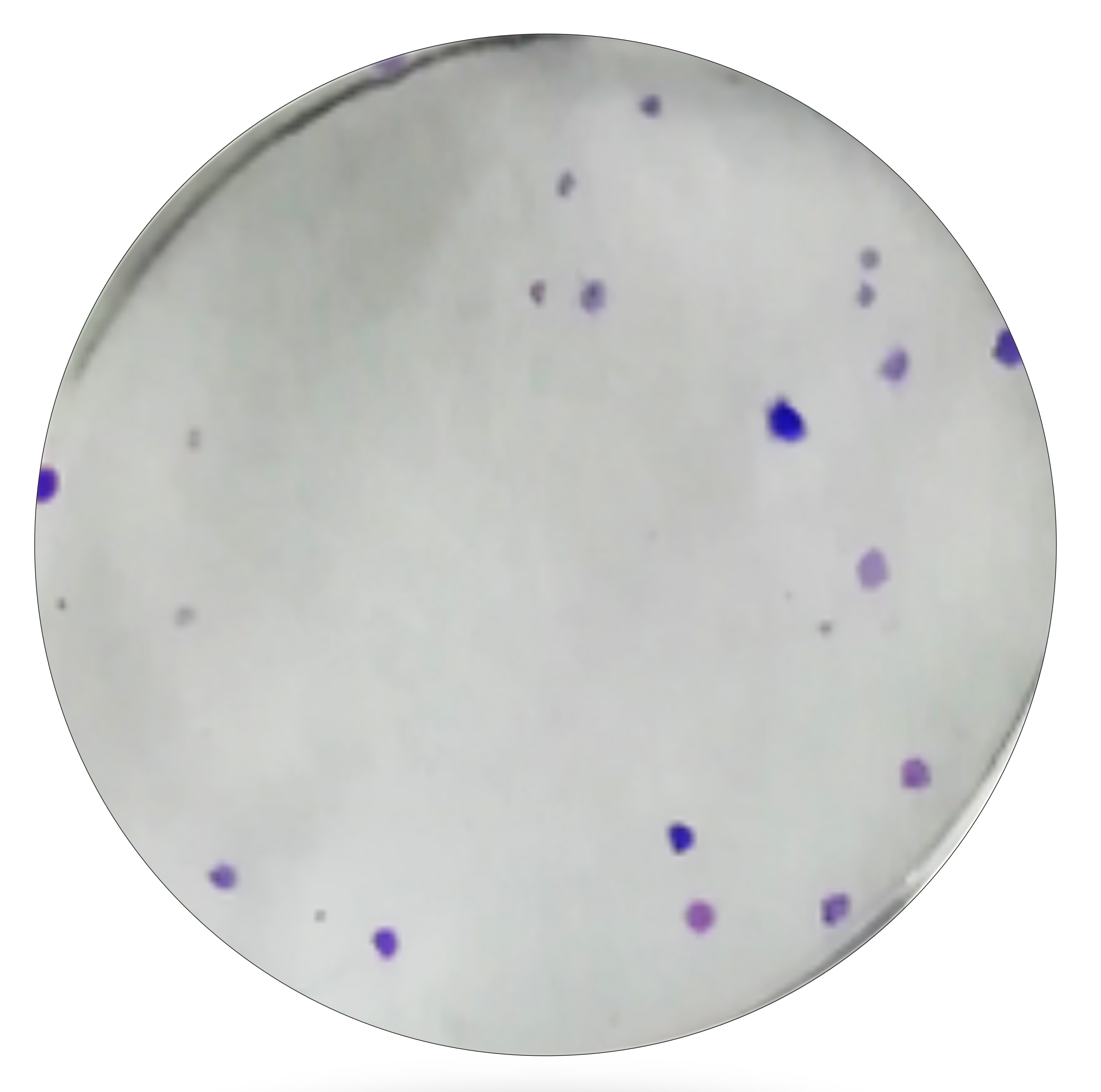

Supplement: Supplementary file 4 [file Image_2.tif]

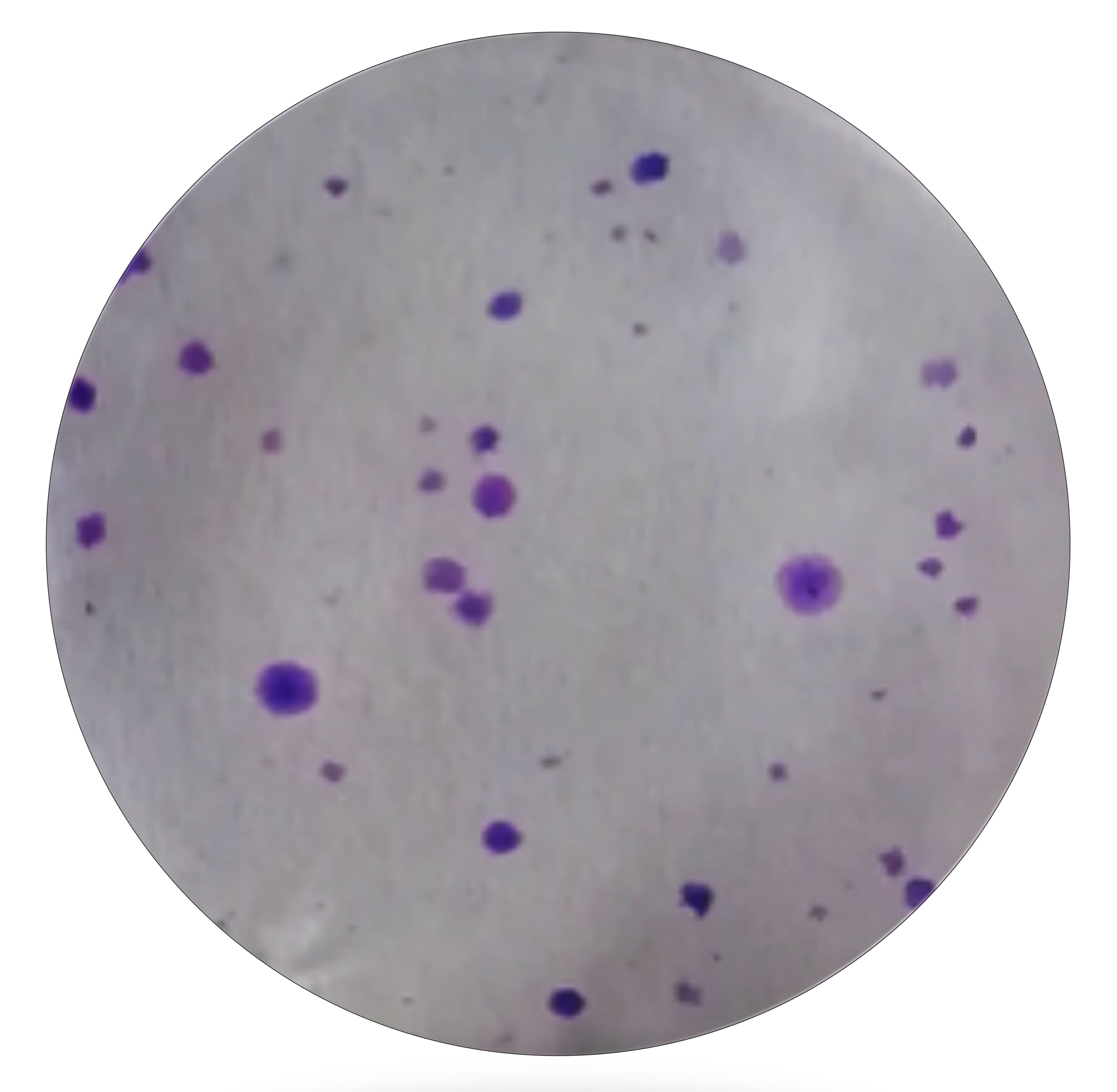

Supplement: Supplementary file 5 [file Image_3.tif]

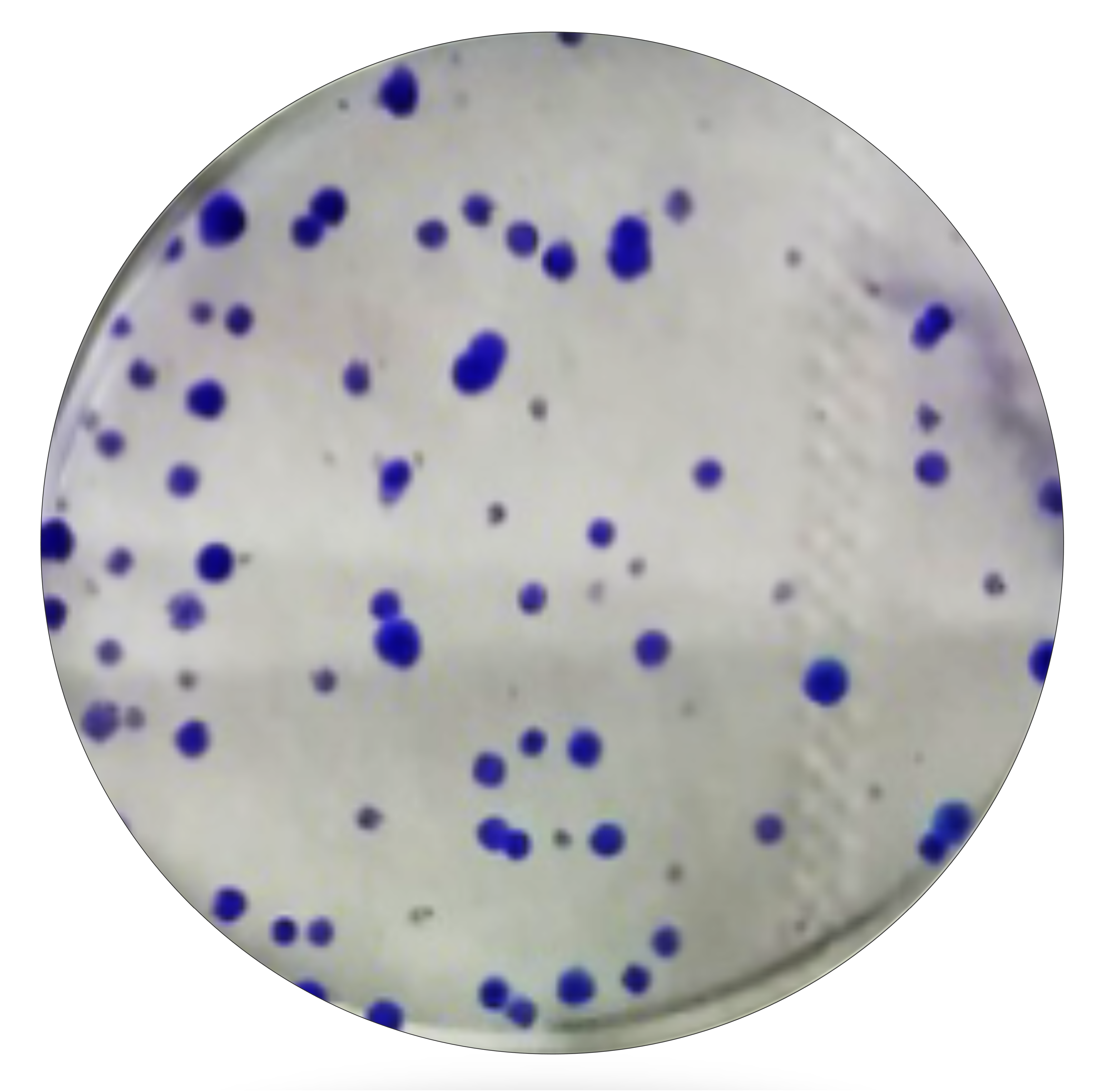

Supplement: Supplementary file 6 [file Image_4.tif]

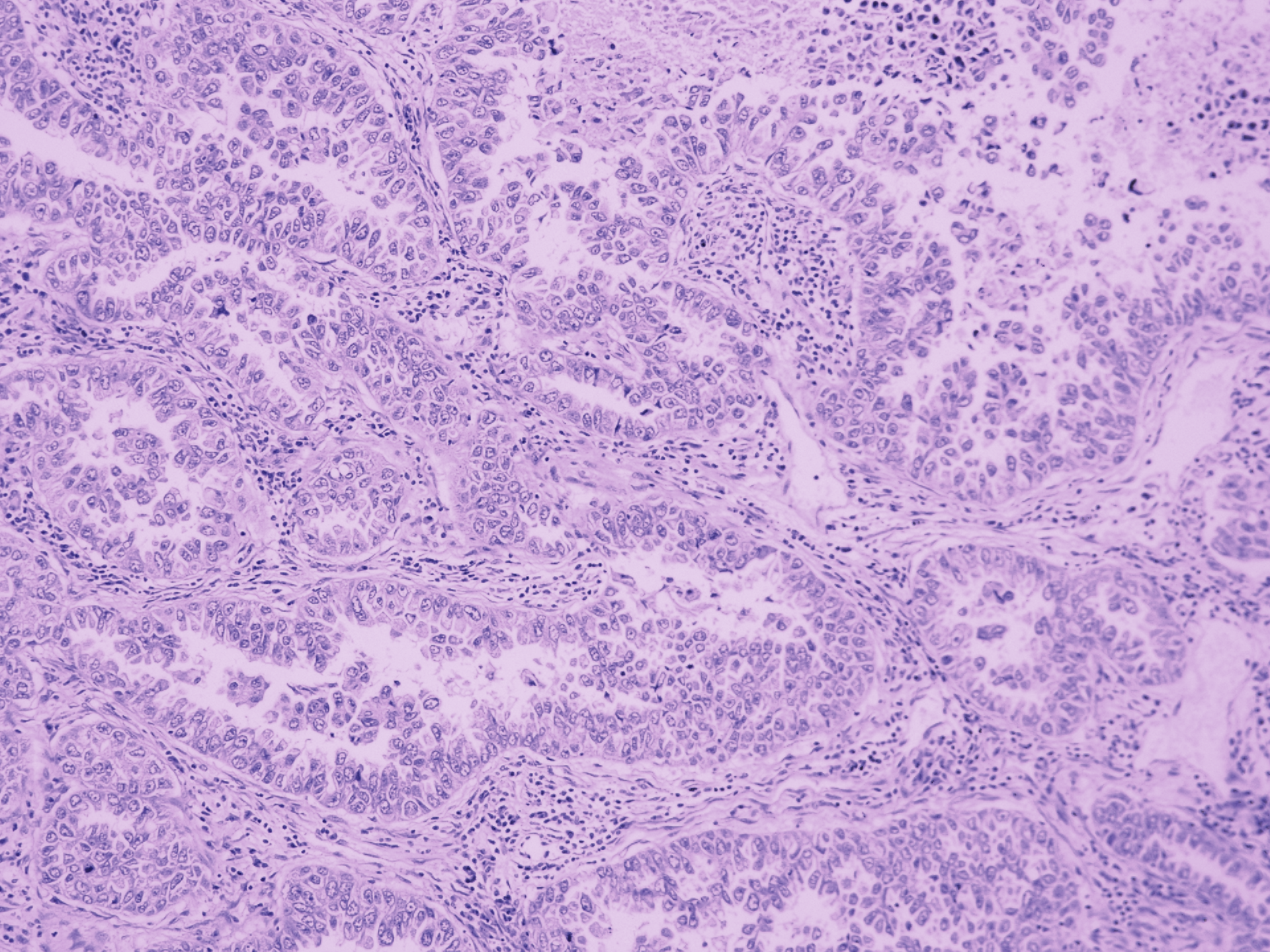

Supplement: Supplementary file 7 [file Image_5.tif]

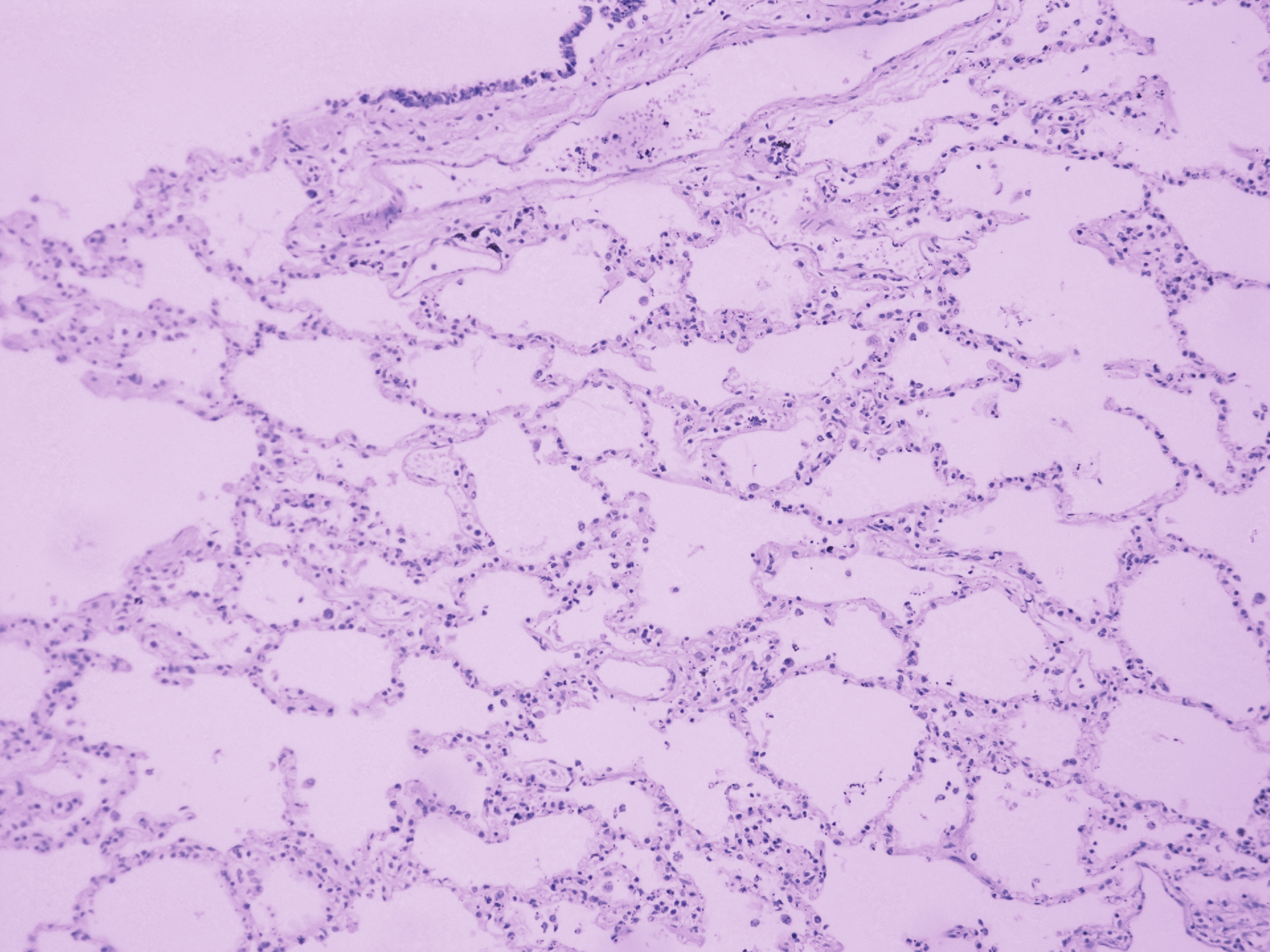

Supplement: Supplementary file 8 [file Image_6.tif]

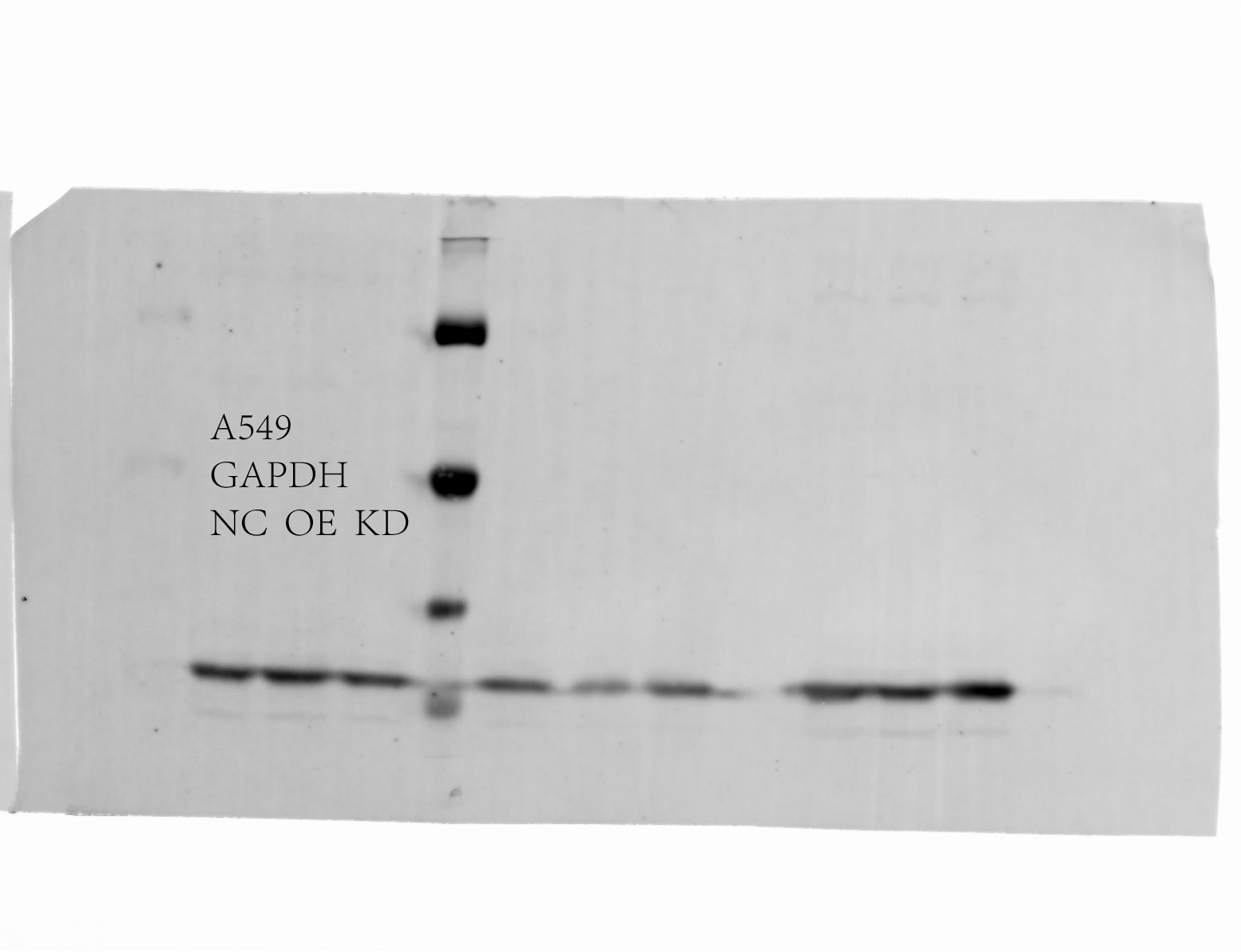

Supplement: Supplementary file 9 [file Image_7.jpeg]

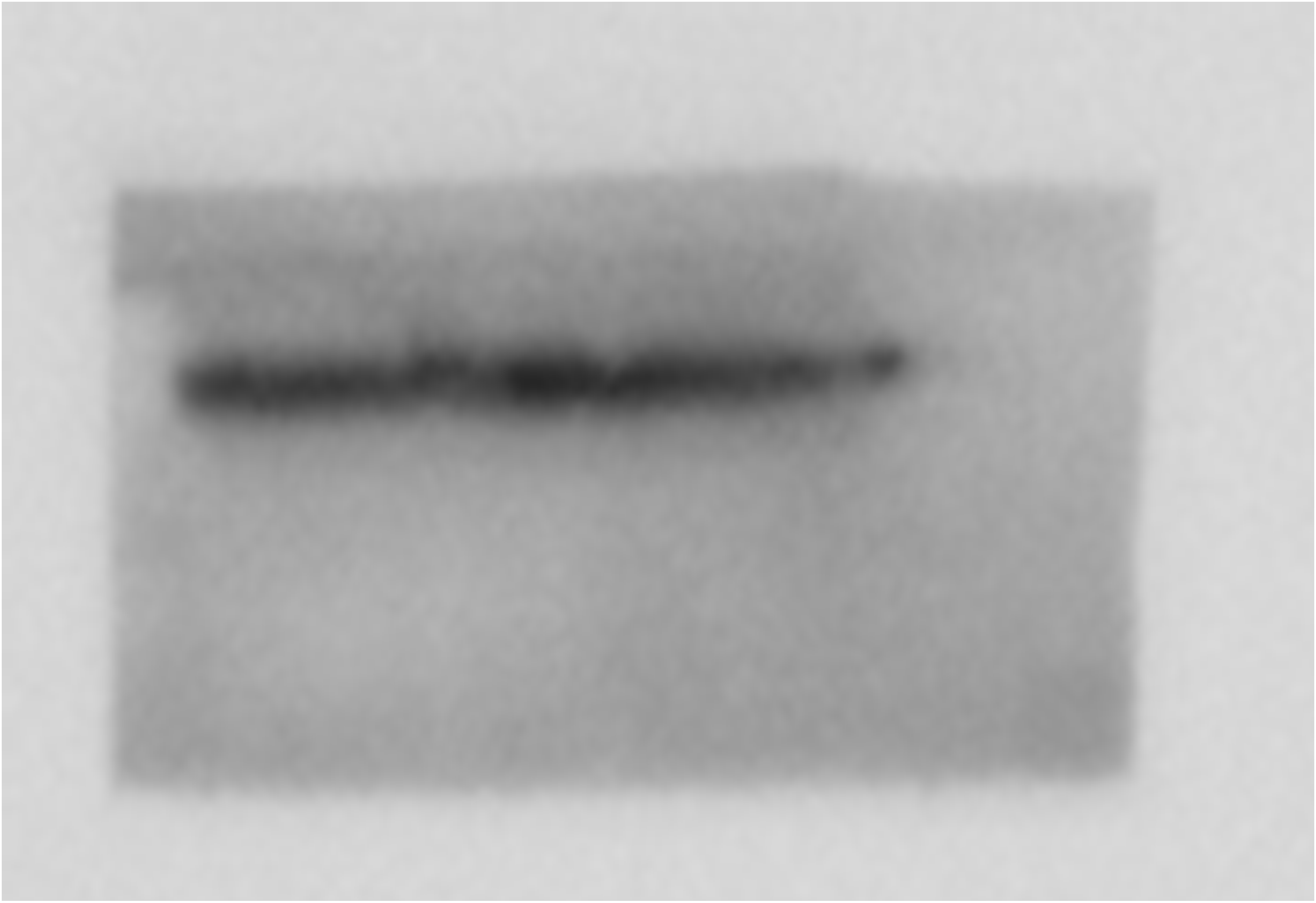

Supplement: Supplementary file 10 [file Image_8.tif]
